# Inflammatory Myofibroblastic Tumor After Receiving Treatment for Non-small Cell Carcinoma

**DOI:** 10.7759/cureus.59359

**Published:** 2024-04-30

**Authors:** Neil C Parker, Prashanth Singanallur, Saif Faiek, John Gao, Peter White

**Affiliations:** 1 Internal Medicine, Southern Illinois University School of Medicine, Springfield, USA; 2 Pulmonary and Critical Care Medicine, Southern Illinois University School of Medicine, Springfield, USA; 3 Pulmonology and Critical Care, Southern Illinois University School of Medicine, Springfield, USA; 4 Pathology, Southern Illinois University School of Medicine, Springfield, USA

**Keywords:** sbrt (stereotactic body radiotherapy), imt, non-squamous cell lung cancer, lung tumor, inflammatory myofibroblastic tumor (imt)

## Abstract

Inflammatory pseudotumor encompasses a spectrum of both neoplastic and non-neoplastic conditions characterized by a histological pattern featuring a proliferation of cytologically bland spindle cells, accompanied by a prominent chronic inflammatory infiltrate. Within this spectrum, inflammatory myofibroblastic tumor (IMT) has emerged as a distinct entity over the past two decades, marked by unique clinical, pathological, and molecular characteristics. Typically affecting the visceral soft tissues of children and adolescents, IMT exhibits a propensity for local recurrence while posing a minimal risk of distant metastasis. They are extremely rare in adults, constituting less than 1% of adult lung tumors. Our patient, a 63-year-old female, has an intricate medical background, encompassing chronic obstructive pulmonary disease (COPD), a previous history of smoking (35 pack-years, quit a year before admission), coronary artery disease, non-obstructive hypertrophic cardiomyopathy, and obstructive sleep apnea. Presenting with a diagnostic dilemma, she recently received treatment for non-small cell carcinoma with radiation therapy, which has evolved into a swiftly advancing case of IMT.

## Introduction

In 1905, the phenomenon of inflammatory pseudo-tumors (IPTs) was initially observed in the orbital tissues of four patients. These growths, coined as "pseudo-tumors," resembled malignancies both clinically and radiologically. Subsequently, in 1939, a subset of these IPTs was identified as inflammatory myofibroblastic tumors (IMTs), mainly found in lung tissues. IMTs are rare mesenchymal neoplasms in the lungs and abdomen, often arising from an excessive inflammatory response. The exact incidence is still being investigated. It is characterized by myofibroblastic spindle cells and an inflammatory infiltrate comprising plasma cells, lymphocytes, and eosinophils [1, 2].

Constituting a minute fraction (0.04 to 0.1%) of all pulmonary neoplasms, IMTs are more prevalent in children compared to adults. Typically benign, a complete cure is achievable through surgical resection. In certain instances, IMTs may exhibit invasive tendencies into adjacent structures, undergo malignant transformations, recur, or even metastasize [1, 2]. This case report highlights an example of a patient with recent non-small cell carcinoma treated with radiation therapy who developed a new, rapidly progressing case of IMTs.

## Case presentation

The patient is a 63-year-old female with a history of chronic obstructive pulmonary disease (COPD), a former smoker (35 pack-years history, quit smoking one year prior to the original presentation), coronary artery disease, non-obstructive hypertrophic cardiomyopathy, and obstructive sleep apnea.

**Figure 1:**
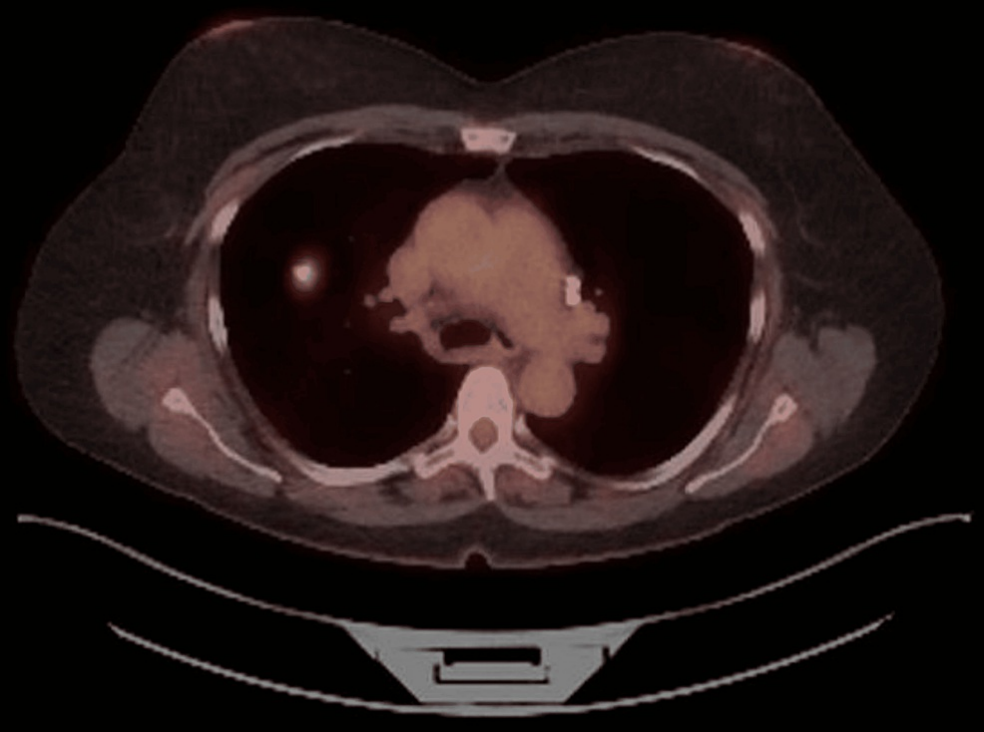
PET-CT scan: hypermetabolic (SUV = 13.1) spiculated RUL pulmonary nodule, consistent with malignancy PET-CT: positron emission tomography-computed tomography, SUV: standard uptake value, RUL: right upper lobe.

The patient underwent a screening low-dose chest CT that showed a new 1-cm spiculated lesion in the inferior aspect of the right upper lobe (RUL). She underwent a PET-CT that showed a markedly hypermetabolic (SUV = 13.1) spiculated RUL pulmonary nodule, consistent with malignancy. It also revealed an adjacent tiny satellite nodule, consistent with an additional site of malignancy (Figure 1). The patient underwent a needle biopsy of the RUL nodule, and immunostains were positive for Napsin-A and thyroid transcription factor 1 (TTF-1), while staining was negative for CK5/6 and P40. These findings were consistent with a diagnosis of non-small cell carcinoma, favoring poorly differentiated adenocarcinoma, clinical stage T2aN0M0. 

**Figure 2:**
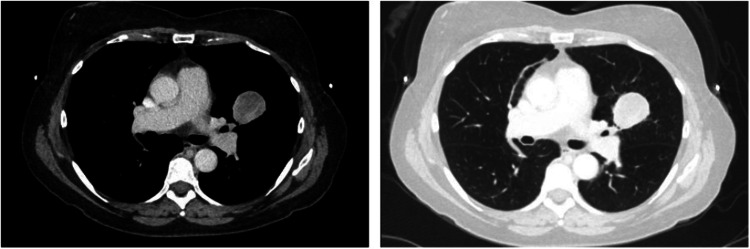
Chest CT scan: a new mass in the left upper lobe that measured 3.2 cm × 3.8 cm

After further discussion with the multidisciplinary tumor board, the patient was deemed not a surgical candidate, given her poor pulmonary function. The patient successfully underwent stereotactic body radiation therapy (SBRT). After completing SBRT, the patient underwent CT scans every six months for the next four years, which showed stable post-radiation fibrosis of the right upper lobe (RUL). The chest CT scans also showed small bilateral pulmonary nodules that intermittently increased and decreased in size, believed to be benign and likely represented previous histoplasmosis fungal infection, which is commonly seen in this part of the country. The patient reported feeling well throughout this period. She continued with the routine screening chest CT scan every six months to one year. Four years after completing her SBRT, her chest CT scan showed a new mass in the left upper lobe that measured 3.2 cm × 3.8 cm (Figure 2).

**Figure 3:**
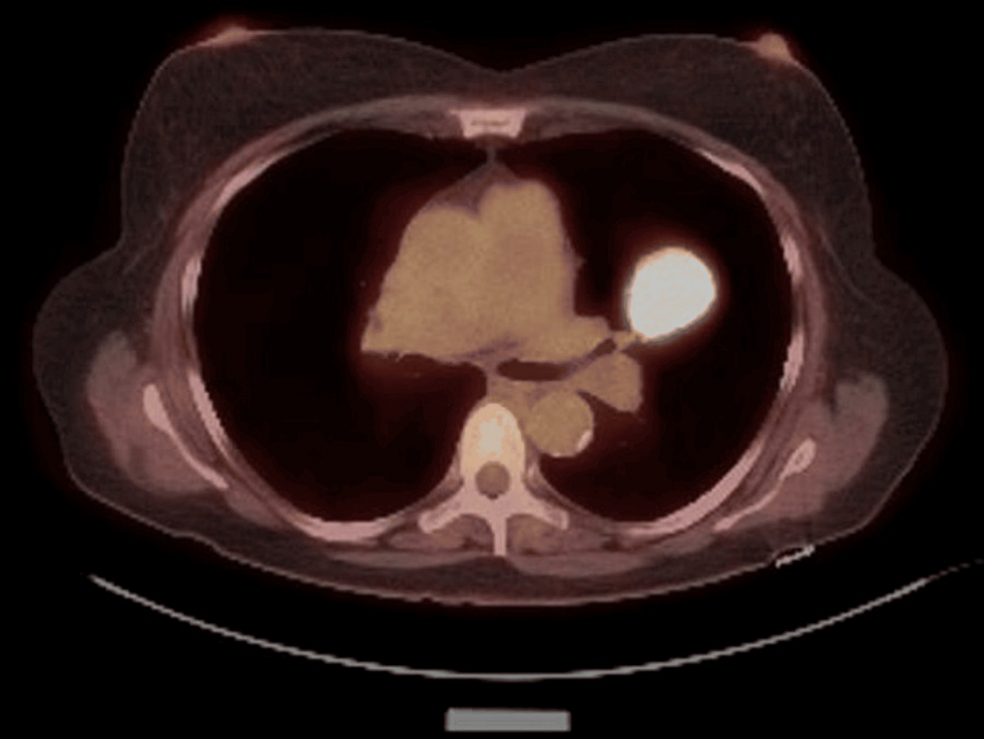
PET-CT scan: a moderately hypermetabolic LUL mass (SUV = 16.2) consistent with bronchogenic carcinoma, with no mediastinal or hilar lymphadenopathy PET-CT: positron emission tomography-computed tomography, SUV: standard uptake value, LUL: left upper lobe.

The patient underwent a PET-CT that showed a moderately hypermetabolic LUL mass (SUV = 16.2) consistent with bronchogenic carcinoma, with no mediastinal or hilar lymphadenopathy. The PET-CT also identified a mildly hypermetabolic (SUV = 3.9) left lower lobe (LLL) nodule, concerning for an additional focus of malignancy (Figure 3).

The patient underwent bronchoscopy and cervical mediastinoscopy. Lymph nodes were sampled from the left and right low paratracheal (L4 and R4) as well as the subcarinal region (station 7), and they were all negative for malignancy. Given the concern for malignancy in the LUL mass, the patient underwent a left upper lobectomy.

**Figure 4:**
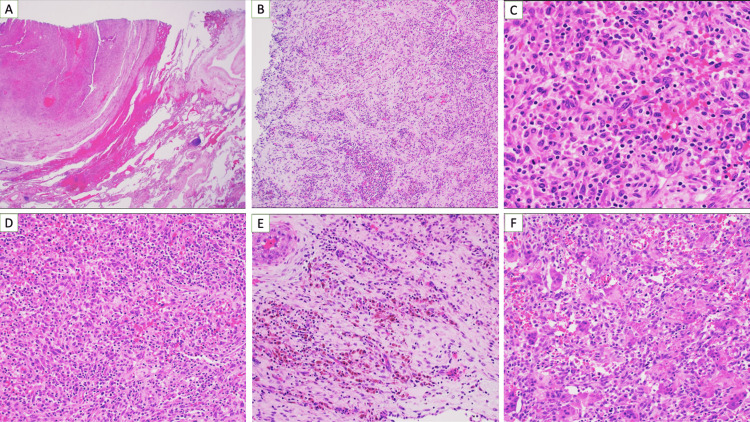
Pathology slides. Immunostains showed that the mass stained positive for CD68, S100, Factor XIIIa, patchy p63, and CD10. It was negative for Napsin, TTF-1, pankeratin, CK8/18, smooth muscle actin, STAT6, CD34, CK5/6, p40, desmin, p53, and ALK-1. Kappa and lambda in situ hybridization showed a polygonal population of plasma cells. A review of the histologic features and the immune profile was most consistent with an inflammatory myofibroblastic tumor CD: cluster of differentiation, TTF-1: thyroid transcription factor-1, CK: cytokeratin, STAT6: signal transducer and activator of transcription 6, ALK-1: anaplastic lymphoma kinase-1.

Histologically, the tumor is composed of fibroinflammatory patterns with variable numbers of plasma cells, lymphocytes, histiocytes, myofibroblasts, multinucleated cells, and hemosiderin (Figure 4).

The patient was seen in follow-up and tolerated the surgery well, with no postoperative complications. Given the biopsy findings, she will continue with regular follow-ups for her known RUL non-small cell carcinoma.

## Discussion

First reported in 1939, inflammatory myofibroblastic tumor is a rare mesenchymal neoplasm that falls under the broader umbrella term "inflammatory pseudotumor," due to its rare incidence of metastasis. Due to their uncertain biological origin, these tumors have also been referred to as plasma cell granuloma, xanthogranuloma, inflammatory myofibroblastic tumor, fibroxanthoma, lymphoid hamartoma, myxoid hamartoma, and fibrous histiocytoma [1, 3]. These tumors most commonly occur in children but have been reported in patients in their seventies. IMT was first identified in the lung but has been found in the abdominopelvic region, retroperitoneum, somatic soft tissues, bone, larynx, uterus, and central nervous system (CNS) [1-3]. As the name implies, these tumors are composed of myofibroblastic spindle cells accompanied by an inflammatory infiltrate of plasma cells, lymphocytes, and eosinophils [1,3]. These tumors have a wide range of presenting symptoms, ranging from no symptoms to cough, hemoptysis, dyspnea, and pleuritic pain when present in the pulmonary system [3].

Treatment for these tumors is as heterogeneous as the tumors themselves. Solitary local disease carries a benign prognosis if resected. Locally invasive, recurrent, or metastatic IMTs present a challenge. Tumors treated with complete resection have only a 2% recurrence risk compared to a 60% recurrence risk after incomplete resection. Multifocal or locally invasive tumors that are unresectable or only partially resectable may be treated medically with either glucocorticoids and/or chemotherapy, immunotherapy, or radiotherapy with limited efficacy [1-3]. Specifically, carboplatin and paclitaxel have been reported to be helpful in some cases, but this response is not generalizable. Similarly, once genetic sequencing of the tumor has been performed, ALK RTK inhibitors such as crizotinib, alectinib, and ceritinib have been noted to be effective in both extrapulmonary and pulmonary IMTs if ALK rearrangement is present, and Crizotinib may be effective if ROS1 kinase fusions are present [2]. IMTs often show fluorodeoxyglucose uptake, and therefore PET scans may be a valuable tool in monitoring for response to treatment in those patients unable to undergo complete surgical resection [3, 4].

This patient had a new case of IMT after being treated with SBRT for non-small cell carcinoma. A literature review did not show a clear correlation with radiation. However, there have been case reports of pediatric patients who presented with pulmonary IMT after undergoing treatment for Wilms tumor [5, 6]. Although the occurrence of the two tumors may have been a coincidence in this patient, there may be a causal link. IMTs are often associated with genetic mutations, which may predispose patients to develop these tumors. It is also possible that the SBRT, which the patient underwent, increased the probability of developing IMT in the future. Further case reports and studies are needed to determine any known correlation between the two.

This particular case is also interesting, given the acuity of the presentation. This patient had a screening chest CT six months prior that showed no mass in that location. The average rate of progression of IMTs is challenging to ascertain, given the rarity of the diagnosis and the frequent lack of symptoms. Some patients may complain of site-specific symptoms, such as chest pain or shortness of breath in pulmonary IMTs. Usually, 15-30% of patients present with "inflammatory" symptoms, including fever, weight loss, or malaise. The non-specificity of these symptoms often delays diagnosis [7], making it difficult to determine the standard growth rate.

## Conclusions

Due to the markedly diverse clinical manifestations of IMT, an ongoing debate persists regarding their etiology: whether they signify an inflammatory reaction secondary to malignancy or originate as a primary inflammatory phenomenon. The absence of definitive diagnostic criteria and standardized management protocols, coupled with the relatively low incidence of IMTs among adults, underscores the challenge faced by many cancer centers in effectively diagnosing and treating these neoplasms. Consequently, there is often a dearth of experience and guidance available in these institutions for managing IMTs. More investigation is still needed to elucidate the risk factors, incidence, and treatment of IMTs.
